# Aesthetic sustainability: relational understanding in conservation research environments

**DOI:** 10.1007/s13194-026-00747-8

**Published:** 2026-06-04

**Authors:** Luana Poliseli

**Affiliations:** Department of Philosophy, School of Social Sciences, University of Manchester, Manchester, England, UK

**Keywords:** Socioenvironmental issues, Biodiversity crisis, Climate change, Aesthetics and arts, Inter- and transdisciplinarity, Environmental aesthetics

## Abstract

Aesthetic experiences are slowly but increasingly gaining attention in the contemporary philosophy of science. However, they remain peripheral in debates about research environments dealing with complex socio-environmental problems. Through a socially engaged and empirically oriented approach, I examine the aesthetic experiences in achievements of understanding in two research environments from the sustainability sciences: the *ClimArtLab Evolving Futures Owing Our Mess* and the *Council of Care*. I further the debate by presenting a relational account of aesthetics in scientific understanding, where understanding is a triadic engagement between subject, object and environment mediated by aesthetic experiences.

## Introduction

Many philosophers of science agree that aesthetics can aid scientists by providing understanding (see Elgin, 1993, Stuart, 2018, Ivanova, 2020). However, how it precisely occurs is not entirely clear. Epistemological debates of scientific understanding take aesthetic considerations as peripheral and mostly focused on historical cases. While recent debates on aesthetics of science bring a refreshing take with contemporary cases, it is still mostly focused on disciplinary practices. In both these pursuits, pluridisciplinary environments dealing with complex and wiked real world problems have been largely ignored. In this paper, I take a step further to fill this gap and incorporate lessons between aesthetics and understanding in inter- and transdisciplinary practices dealing with complex socio-environmental problems.

Through an empirically-oriented and socially-engaged approach, I examine the aesthetic experiences in two research environments in the sustainability sciences: the *ClimArtLab Evolving Futures: Owning Our Mess*, a collaboration between arts, science, and the humanities focusing on Climate Change; and the *Council of Care*, a transdisciplinary research team focusing on knowledge co-production with traditional communities for biodiversity conservation. To further contemporary aesthetics debates from the advantage point of providing understanding, I present a relational aesthetic account of scientific understanding. According to this account, scientific understanding is *(i)* a process of embodied-enactement of scientific knowledge facilitated by aesthetic experiences, *(ii)* which is constantly shaped by the relationship between the subject, object, and the environment.

This article is structured as follows. Section two identifies some directions in which aesthetics is embedded in accounts of scientific understanding and addresses key challenges faced by contemporary accounts of understanding. This section serves a diagnostic function: it maps the broader theoretical terrain in order to demonstrate its limitations and how I plan to overcome them. Section three explains what is accounted for as research environments beyond disciplinary traditions. I discuss the relation of aesthetics and understanding in the *ClimArtLab* and the *Council of Care*, respectively. Section four contends for the plurality of aesthetic experiences to achievements of understanding by introducing a *relational aesthetic account of scientific understanding*, where I discuss its connection to previous frameworks, the role of environment, and its relational features. Finally, section five presents conclusion remarks.

## Directions of aesthetics in scientific understanding

Scientific theories, models, and experiments are uncontroversially often subject to aesthetic assessment (Elgin, 2020). They can motivate scientists to study a phenomenon, guide their activities, and even shape their attitudes regarding the truth of a theory. Aesthetic experiences in science can have, therefore, several epistemic advantages (Stuart, 2018) such as empirical adequacy, heuristics in the underdetermination of a theory, relations to truth, and so on (Ivanova & French, 2020)^1^. While this strengthens the idea that aesthetic experiences can serve as conditions for understanding (Kosso, 2002), the relationship between aesthetics and understanding is not entirely clear.

In the epistemological debates of scientific understanding, aesthetic experiences, whenever acknowledged, are discussed according to singular aesthetic properties related to understanding. This leads to the assumption that aesthetic properties can only partake in the process of understanding through an auxiliary role. However, understanding scientific understanding *vis-a-vis* aesthetic experiences gives the advantage of grasping and dealing with the heterogeneity of scientific practices. On these grounds, and although debates on scientific understanding are longer than the scope of the paper, I will only focus on accounts of scientific understanding that engage with aesthetic properties. Similarly, and to narrow down the growing literature in aesthetics of science, I will only engage with aesthetics of science literacy^2^ that draws upon the epistemic advantage of providing understanding. In a non-exhaustive fashion, in this section, I identify distinct (albeit interconnected) directions in which aesthetic features have been connected to scientific understanding in epistemology and aesthetics of science (as in: unification, visual, pictorial, embodied, imaginative, thought experiments, and processual), and present some challenges of these accounts.

### Understanding through unification

According to Ivanova (2017a, 2020), an interesting link between understanding and aesthetic values can be found in Poincaré’s aim of science. According to this account, the goal of science is not truth but rather understanding of how phenomena are related. Aesthetic values such as simplicity and unity are regulative ideals linked to this ultimate aim of science: understand the relations between phenomena. For Poincaré, beauty is experienced when one has grasped how different and apparently disconnected phenomena are unified. Poincaré reduces beauty to the simplicity, harmony, and unity of theories. He argues that these values persist as ideals of science rather than being subject to time and fashion and are conditions of thinking. These accounts are instructive in overcoming many of the objections noted above regarding the link between aesthetic values and truth and in establishing the epistemic role of aesthetic values (ibid.).

### Understanding through visualization

The ‘contextual theory of scientific understanding’ (De Regt & Dieks, 2005; De Regt, 2017) elaborates on the history of science, specifically the history of physics, to assert that understanding is only achieved through intelligible theories. The more a theory is intelligible, the bigger the chances to understand it. It focuses on the idea of variations in standards of intelligibility in scientific practice, taking into consideration the contemporary and historical practice of science. In this sense, it does not claim a status of exclusiveness and immutability because of the importance of changing contexts. Although it also acknowledges unification as a tool for understanding, the contextual account asserts that scientists usually adopt other conceptual tools such as causal reasoning, mathematical index, visualizability, and visualization to improve the intelligibility of a theory. Visualizability here is a theoretical quality that helps enhance intelligibility, while visualization is regarded as a useful guide to achieving scientific understanding. De Regt (2014), elaborates on concrete examples of visualization in twentieth-century theoretical physics to show that visualizable theories are often regarded as more intelligible than abstract ones, as many scientists prefer visual reasoning in the construction of explanations.

### Understanding through pictures

Meynell (2018, 2020) furthers De Regt’s by elaborating on a pictorial account that considers having understanding of non-factual things. According to it, the characteristic content of understanding is pictorial rather than propositional. ‘Pictures’ would possess main features that are key to exemplifying the cognitive processes and values characteristic of understanding: their affinity to visual experiences via two-dimension and their representational role that serves as props for imagination. The epistemic flexibility of pictures would virtuously allow this account to embrace unificatory, mechanistic, and pluralist views of understanding since “to understand something is to be able to see the parts in relation to each other and as they relate to the whole” (Meynell, 2020:58). The distinction between the contextual account and the pictorial account is that, for Meynell, visualization is deemed a necessary condition for understanding, whereas for De Regt, it is just one of many available tools. One challenge in using images to convey understanding is that if the theoretical content does not align with the form of visual representation, it is more likely to create misunderstanding rather than clarity (see Greehalgh et al., n.d.) for examples related to visuals and illusions in public health; and Murphy (2023) for content and form on thought experiments).

### Understanding through embodiment

Leonelli (2009) provides an account of scientific understanding via modelling practices and skills in contemporary biological research. According to her, in biological research environments, there are at least three types of understanding at play: theoretical, embodied, and integrated. In *theoretical understanding*, the understanding of biological phenomena would rely on theoretical commitment and skills, e.g. population biology. In *embodied understanding*, the central aspect would be to acquire performative skills that would be useful to explore and intervene in the phenomena, e.g. biomedical sciences. And in *integrated understanding*, both theoretical and embodied understanding are equally important to understanding a phenomenon. Although Leonelli does not present any aesthetic properties on her account, her framework will be relevant for late debate as it underscores that each type of understanding will be adequate for distinct research contexts since scientists are subject to their research settings and goals.

### Understanding through imagination

Focusing on the role of imagination in achievements of understanding, Breitenbach (2019) argues against the heterogeneity of imagination, and proposes an unified account of the mental activities of imagination. According to her, there is an unexpected connection between aesthetic experience in science and the scientist’s aim of understanding, which is the same kind of imaginative activities that lie at the basis of the aesthetic experiences in an artistic context. Whether one is experiencing a piece of art or experiencing a scientific activity, one uses imagination to explore interpretations of the abstract concepts that a theory or a piece of art contains. One imaginatively represents an individual phenomenon that may be unified under the concepts that are being interpreted. It is the virtue of creating a narrative through reflexive imagination. These imaginative activities help to see how the theory can be developed further and what additional problems it might help to solve. According to her account, imaginative activity is a further part of the ability to achieve and deepen understanding. Imagination, in this sense, would be a *sine qua non* of science (Stuart, 2017). A similar argument is also made by Tateo (2020) where imagination is considered a higher mental process.

### Understanding through thought experiments

Rather than directly intervening in the world, thought experiments occur in the realms of imagination, as a laboratory of the mind (Brown, 2011). Although thought experiments are embedded in imagination, the extent of debate surrounding them in the aesthetics of science warrants a specific spot. Thought experiments can aid in supporting, elucidating, or challenging theories, as they typically involve thinking about an imaginary (or fictional) scenario and assessing the possible outcomes if they were to occur in reality (Murphy, 2020; for fiction see Frigg, 2008, 2010; Elgin, 2014; Currie, 2016; Knnuttila 2021). They not only have the potential to increase understanding, but also if open-ended and goal-directed can encourage scientists to perform certain cognitive actions, which can be repeated and reinforced to build new abilities and skills (Stuart, 2018). An example of how thought experiments relate to understanding is explained on Poliseli (2020) processual account.

### Understanding through processes

In assessing an interdisciplinary ecological research practice, Poliseli (2020) elaborated an account where understanding is a gradative process that relies on degrees of understanding, imagination, and knowledge selectivity. According to this account, understanding is achieved through gradual progressions that are directly related to the complexity of the information into consideration. Part of this process depends on the use of imagination (through mental models and thought experiments) and on the capacity of the scientist to filter the information relevant to explain the phenomenon in question. I will unpack.

According to this account, scientific understanding is achieved in degrees (an idea consistent with Kvanving (2003), Baumberger (2019), and others), and depends on epistemic skills, imagination, and knowledge selectivity. First, degrees of understanding are directly related to the complexity of the phenomenon: the more complex, the more gradually understanding is achieved. Second, in certain contexts, either theoretical knowledge or practical skills alone may suffice to generate understanding, aligning with Leoneli’s (2009) notion of *integrated understanding*. However, when dealing with a particular complex phenomena or theories, resorting solely on theoretical knowledge or a specific skill set may be insufficient. In such cases, additional cognitive tools are required, in line with De Regt (2017) *contextual account*. In the ecological research environment examined by Poliseli (2020), imagination (i.e. thought experiments) served as a valuable resource during model-building and acted as central mediator in the achievement of understanding. This aligns with discussions on the role of imagination in scientific understanding found in Breitenbach (2019), Todd (2020), and others. And third, a key feature of this account involves *knowledge selectivity*. Achieving understanding is not related to accumulating knowledge, in other words, having understanding is not a matter of having a larger sum of knowledge. A scientist should be apt to judge which information or evidence is relevant to explain certain phenomena. The more understanding of a phenomenon a scientist acquires, the better equipped they will be to select relevant scientific knowledge and exclude irrelevant ones (Poliseli, 2020).

### Facing the challenges in contemporary accounts of understanding

Although there is an increasing effort to account for aesthetic factors in scientific understanding, as the ones underscored by the previous section, some challenges still remain.

First, by grounding their analysis in disciplinary scientific practices, these accounts often miss how understanding is shaped within multidisciplinary or interdisciplinary contexts. In research environments addressing complex and wicked societal issues such as climate change, biodiversity crisis, sustainability, rewildering, etc.; aesthetics and epistemic practices intersect in more complex ways. The knowledge co-created and generated from these practices are usually focused in deriving empirical deliverables that will have direct societal impact, either in developing public policies, either in generating public understanding of science, and so on. Regardless, achievements of understanding within these contexts are also influenced by understanding the impact of scientific knowledge in society. Therefore, to address this challenge, I will consider that the production of scientific knowledge is situated (Haraway, 1988, 2016) hence *debates of scientific understanding should also acknowledge that in these settings understanding is not only scientifically contextual*, as defended by De Regt (2017), *but also dependent upon other academic and cultural traditions*. Thus, *the knowledge being produced*,* the aesthetic experiences being engaged*,* and the understanding being achieved will happen in relation to these practices*,* that is intertwined with the societal problem at stake*. The notion of ‘aesthetic cultures’ developed in Currie (2023a) adds to the reflection that aesthetic judgments in science are influenced by the specificities in which scientific objects and processes are regarded by scientists and society. Distinct aesthetic judgments are justified because of specific education and disciplinary contexts, and not due to historical cases of success such as McAllister’s (1996) notion of aesthetic culture would argue (ibid.).

Second, when scientific understanding is framed solely in terms of propositional knowledge that requires complex theories, models, and expert-level explanations, it sets the stage to only subject-matter experts to fully understand the object, which is understandable considering the contextual characteristics and aims of scientific practices. However, when targeting socio-environmental issues, if the desire is to cultivate citizens with the agency and awareness to act toward sustainable futures, a fundamental point should not be overlooked: *anyone should be able to understand complex scientific information*,* either in disciplinary*,* multidisciplinary and transdisciplinary contexts; academic and non-academic settings.* Therefore, scientific knowledge should also be communicated in ways that are intelligible to non-scientists and non-experts embedded in distinct research environments. To address this challenge, this paper calls for thinking scientific knowledge beyond just propositional or pictorial representations in such a way that understanding scientific information is inclusive to a variety of contexts and a variety of audiences (see Poliseli & Caniglia, 2024).

Third, by focusing on singular aesthetic features two problems arise: (i) it misses to capture the broader range of aesthetic dimensions at play in scientific work. As a result, they offer only a partial picture of how aesthetics contributes to provide understanding; And (ii) by underscoring the aesthetic properties of scientific objects (e.g. visualization, pictures, models), they neglect a crucial component of aesthetic engagement: its dialogical and experiential nature.

To address problem (i), the current blind spot concerning the heterogeneity of aesthetic engagements, I will, where appropriate, introduce contemporary debates on the aesthetics of science to help bring up to speed the inherently heterogeneous nature of the aesthetics in science. Aesthetic considerations are pervasive throughout scientific activity, and as Ivanova ([Bibr CR46][Bibr CR48], 2023, Ivanova et al., 2024) has extensively demonstrated in her work, aesthetic values enter science on at least three different levels: the objects of study, the products of science, and the scientific practice. Building on this, I propose that aesthetics also play a role in three additional domains: (1) research teams collaborations, especially within inter- and transdisciplinary contexts; (2) science communication, including SciArt, DataVisualization, and other forms of public engagement; and (3) the science-policy interface, such as synthesis reviews, evidence-based policies, and decision-making processes (see Greenhalg et al. under review). Therefore, to properly assess how aesthetic and scientific understanding interrelate, I must account not only for the diversity of contexts in which these engagements occur but also for the variety of aesthetic experiences *per se.* In this regard, Poliseli (2024) perusal on distinct dimensions of aesthetic engagements (as emotional, artistic, methodological, intuitive, and sensorial) will be of value to later developments (see Table 1).

**Table 1:**
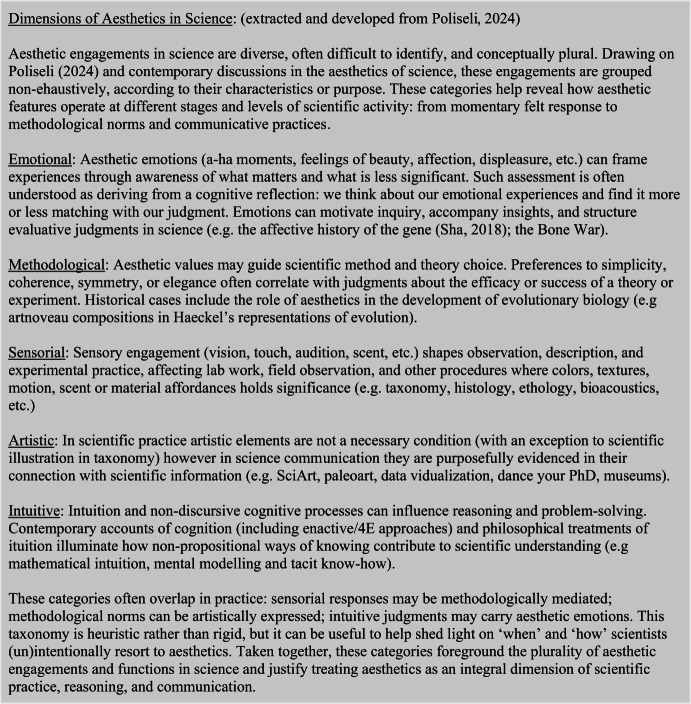
Diversity of aesthetic engagements in science

To address problem (ii), namely, that many existing accounts reduce aesthetic engagement to static properties of theories or models, I argue that such approaches overlook the dialogical and experiential nature of aesthetic experience. More specifically, they tend to frame aesthetics in terms of the object (e.g. a theory or model) and the agent (e.g. scientist), neglecting the broader context in which these experiences occur. In disciplinary settings, Currie’s (2023b) insight that the relationship between aesthetics and knowledge is psychological and contingent upon scientific practices offers a useful starting point for understanding how aesthetics contributes to scientific understanding. However, in inter- and transdisciplinary contexts, particularly when addressing specific socio-environmental challenges, the environment itself plays a critical role in shaping aesthetic responses. To grapple with this complexity, *I propose moving beyond the conventional object-agent model to an object-agent-environment framework* (Brady, 2003). *In these expanded contexts*,* aesthetic experience is co-constituted by the subject*,* the object of inquiry*,* and the surrounding environment.* Crucially, in dealing with socioenvironmental issues, our relationship with the environment, understood broadly to include both natural and social dimensions, is a vital component in how aesthetic responses emerge (Carlson, 1981, 2005).

Lastly, the limitation of the abovementioned accounts of scientific understanding lie in their nuanced focus on isolated aesthetic features, which overlooks the broader scope of aesthetic experiences in scientific understanding, especially as it unfolds across diverse research environments. By reducing the diversity of aesthetic experiences to singular features, they fail to recognize that the strength and epistemic value of the aesthetics in science derive from their inherent diversity, adaptability, and capacity to take on multiple forms. This becomes particularly evident when we consider how aesthetic experiences provide understanding across distinct research environments.

## Research environments beyond disciplinary traditions

Research environments are inherently heterogeneous, they vary across scientific disciplines, historical periods, research schools, and even among individual researchers or teams (McAllister, 1996). Scientific knowledge involves more than just hypothesis testing and communicating results, it is also a collaborative process of knowledge production that depends on the coordinated efforts of diverse, skilled groups working towards common epistemic goals (Currie, 2019). Although no unified model fully captures this diversity of practices, I adopt the perspective of Oliveira et al. (2023), *who conceptualizes scientific practice as an ecological-enactive co-construction of scientific knowledge. In this view*,* the interaction between the subject and object is mutually constraining*,* leading to the emergence of new ways of acting and understanding that enrich human cognitive embodiment*.

As Brady et al. (2018) suggests, during aesthetic experiences, the relationship between subject and object gives rise to properties, qualities, values, and judgments that are typically associated with sensory, imaginative, and emotional dimensions. Given that aesthetic features are pervasive in scientific practices, it is important to consider how aesthetic experiences arise in distinct contexts. Thus, the underlying rationale throughout this paper is that *aesthetic experiences in science are not shaped only by the subject-object relationship but also by the distinct characteristics of specific research environments*^3^.

In the context of the current socioenvironmental crisis, scientists are increasingly compelled to develop creative solutions to complex and wicked problems that cannot be adequately addressed through single-disciplinary approaches alone. For this reason, *I define research environments broadly*,* encompassing not only disciplinary settings but also inter- and transdisciplinary contexts*. I adopt Tress et al. (2005) definitions of research collaborations: interdisciplinary projects integrate knowledge from multiple disciplinary fields to create new understandings and address shared research goals or real-world problems, while transdisciplinary projects involve collaboration between academic researchers from diverse and often unrelated disciplines alongside non-academic participants to co-produce knowledge or solve specific issues. A key feature of these research environments is the interaction among experts from diverse domains, where mutual constraints arise due to epistemic asymmetries. These asymmetries stem, among other factors, from differences in methodologies, conceptual frameworks, intellectual resources, and research practices (Andersen & Wagenknecht, 2013; Poliseli & Leite, 2021).

Although one can infer a heterogeneity of aesthetic experiences across distinct research contexts, and despite growing interest in how inter- and transdisciplinary practices unfold (Poliseli & Caniglia, 2024), empirical and theoretical investigations on the aesthetic experiences and achievements of understanding in these research environments remains limited. Given that the connection between science and the wider world is crucial for understanding both how knowledge is generated and the nature of scientific inquiry itself (Currie, 2019), in the following sections, I explore distinct research environments engaged with conservation and sustainability to assess the types of aesthetic experiences in place and how they can significantly contribute to the broader understanding, theoretical underpinnings, and epistemic values of aesthetic experiences in science.

Furthermore, to investigate how aesthetic experiences inform scientific understanding and how they relate to distinct research environments, I will specifically focus on two transdisciplinary research teams: the *ClimArt Lab* and the *Council of Care*. The first involves a co-creation of an art-science-humanities project aimed at communicating Climate Change. The second centers on knowledge co-creation between biologists, traditional communities, and stakeholders focusing on biodiversity conservation. While describing these research environments in the next sections, I will examine the aesthetic experiences engaged in these practices and gradually discuss its connections with the epistemic advantage of fostering understanding. Due to reasons of space and the focus of this paper, to underscore the plurality and diversity of aesthetic experiences in relation to understanding across research environments, these case studies will be discussed concisely. However, references to more detailed sources are provided for further exploration.

### Understanding & communicating climate crisis

The first research environment, *ClimArtLab Evolving Futures: Owning Our Mess* (2020–2021), was a transdisciplinary collaboration between 2 artists, 1 climate scientist (glaciologist), 2 philosophers of science, 1 sustainability scientist, and 1 art historian. It was initiated by the think-tank artEC/Oindustry and jointly developed with the Konrad Lorenz Institute for Evolution and Cognition Research (KLI), Austria. *Evolving Futures* was an experimental space that combined arts, sciences, and the humanities to co-create interventions to communicate climate change. Its main goal was to find ways to generate intrinsic motivation and agency for climate awareness and behavioural change, while also relying on understanding complex relationships connecting behaviours and the climate emergency through the water-energy-food nexus. The process of knowledge co-creation in the *ClimArtLab* led to two artistic interventions HOMONEXUS and GLACIER NEX US.

HOMONEXUS was a participatory textile installation combined with a Fandango soundtrack performance. The traditional craft of embroidery was used as input for collective meditation, where the installation embraced an embodied and collective approach to cognition and motivation concerning the emotional challenges that climate change presents, with a particular focus on the activity of a collective embroidery through a QR code pattern, leading the audience towards information of Climate Change, Water-Energy-Food nexus available through the *Evolving Futures* website^4^. The installation and collective embroidery were accompanied by a Fandango soundtrack of *Son Jarocho*, which is a traditional type of music and dance from Mexico that promotes intergenerational encounters among individuals, fostering a feeling of social bonding among the participants. GLACIER NEX US was a performance that consisted of a dialogue, an online meeting, between a melting glacier and a glaciologist, which enacted care and despair between humans and more-than-human entities through the caring glacier-glaciologist relationship, while at the same time contrasting it with the despair of humankind towards non-humans, in this case the glacier. A crucial aspect of this performance was the appeal of the personified glacier. The embodiment of a glacier through ‘virtual identity disruption’ catalysed a kind of environmental imagination that put into perspective human-technology-nature relations. The dramatic end of the dialogue (similar to when someone loses connection during a virtual call) allows the imagination to access a (not so) fictional scenery of a society that thrives in its technological dimension while nature ceases to exist. For more details on the performances and the workshops for the co-creation of these artistic performances, see Poliseli and Caniglia (2024).

For this research environment, I draw lessons from Poliseli and Caniglia’s (2024) in-depth analysis of this collaboration. These authors brought to light how inter- and transdisciplinarity reasoning took place, and through a detailed description of this process, it was possible to identify distinct aesthetic features at play, enabling understanding of climate change, such as c*omplexity*,* imagination*,* creativity*,* music*,* dramaturgy*,* metaphors*, etc. For reasons of space, I will focus on *complexity* and *metaphors*.

To make scientific complexities accessible to society, science communication simplifies knowledge through diagrams, mechanisms, and graphs; an approach consonant with Meynel’s (2020) claim that understanding is pictorial rather than propositional. For scientists, this is typically regarded as an epistemic virtue, since they can provide understanding through visualizability (De Regt, 2017). However, for the artists in *Evolving Futures* the opposite seemed to occur. When explaining climate change, the glaciologist employed several graphs to showcase fluctuations of temperature and the rise of ocean and sea levels over time. While for a climate scientist graphical tools are the conventional way to convey planetary and historical processes, the artists experienced such “diagramification of knowledge” as evoking cognitive dissonances rather than understanding, standing in opposition to De Regt’s (2014, 2017) and Meynel’s (2020) defence of the visual and pictorial dimensions of scientific understanding.

Knowing that climate change is a complex phenomenon, meaning that the causal underpinnings and systemic dimensions are not easily captured (Hulme, 2014), the idea of simplifying these complexities into a picture, or diagram seemed reductionistic to the artists’ eyes. Although the collaborators agreed on the intentions of addressing the climate emergency through water-energy-food nexus thinking, they also agreed that it only conveyed partial aspects of this phenomenon. Thus, why not understand nexus and climate change through the embeddedness of its complexity in its systemic way?

*Complexity* is usually experienced as a mixture of order and chaos, allowing explorations to take place as well as pattern findings (Birkin, 2010). Even though *symmetry* (i.e. a geometric property that affects people’s aesthetic experience as the preference for symmetrical patterns (Huang et al., 2018) is usually introduced as a counterbalance to *complexity* (Papadimitriou, 2020), the collaborators preferred to refer to a dichotomy between *simplicity* and *complexity*. For them to understand climate change was a matter of understanding its systemic and dynamic nature and, when necessary, reducing and simplifying the information to make it intelligible, showcasing an alignment with Poliseli’s (2020) notion of understanding, where the more understanding of an object (or phenomenon), the better equipped they will be to select relevant knowledge and exclude irrelevant ones.

During this collaboration, the participants also deployed *metaphors* to communicate and instantiate their rationale. Metaphors are usually considered to be a conceptual phenomenon and a linguistic (or non-linguistic) device where one thing is represented as something else (Lakoff & Johnson, 1980). It is often the interaction between two distinct domains, when features from one domain are mapped onto an object or a concept in another domain, creating a new meaning connecting both domains. In this sense, metaphors enable understanding and experiencing one thing in terms of another (ibid.).

In this collaboration, the HOMONEXUS performance adopted embroidery as a metaphor for the interconnectedness of water-energy-food nexus. The act of sewing was conceived as an invitation to slow down: Slow the food chain, slow the energy consumption, slow the water waste. This proposal resonated with Ingold’s (2016) metaphor of a world interwoven and interconnected through lines, a framework introduced by one of the philosophers. For both the artist and the philosopher of science, the idea of collectively sewing a QR code exemplified how embroidered lines could function not only as metaphors but also as embodied enactments of the interconnectedness within the water-energy-food nexus. The process invited the audience to reflect through practical, sensorial engagement. The QR code represented connectivity, both within the nexus and in contemporary society. In this setting, climate change is also understood through the practical, embodied experience of sewing the water-energy-food nexus.

### Understanding & deliberating biodiversity crisis

The second research environment, the *Council of Care*, was a transdisciplinary practice involving biologists, social scientists, traditional communities, and local stakeholders. Although developed within the project Global Epistemologies and Ontologies of Science^5^ at Wageningen University, Netherlands, its implementation took place in the fishing village Siribinha, Bahia, Brazil.

The *Council of Car*e is a methodological tool for knowledge co-creation in transdisciplinary settings focusing on biodiversity crisis (Ressiore & De la Rosa, 2025). *It is a mock decision-making process where participants enact beings of nature and collaborate to propose solutions to specific socioenvironmental problems.* In this council, the ecological issue was the 2019 oil spil along Brazil’s Northeast coast which struck beaches, mangroves, and estuaries. The unprecedent spill went largely unnoticed as it arrived, leaving authorities and local communities uncertain about its origin, extent and trajectory, becoming the worst environmental disaster in Brazilian coastal history. The fishing village Siribinha was among the affected communities, and the *Council of Care* was tailored retroactively to address this event. For specific details of the methodological design and implementation of the *Council of Care*, see Ressiore & De la Rosa (2025).

The *Council of Care* was designed by combining principles from the Theatre of the Oppressed (Boal, 1991), and the Parliament of Things (Latour, 2018), grounded in the theoretical framework of more-than-human care (Haraway, 2016, De la Bellacasa, 2017). Its thought-provoking aspect lies in the composition of its members: both humans and non-humans, such as policymakers, rivers, trees, environmentalists, fisherman, crabs, sharks, mangroves, ocean, bird, and so on. By giving voice to elements and beings of nature, the *Council* invited participants to move from the question “What do I need as an individual?” toward “What is possible for all?” (Ressiore & De la Rosa, 2025). Since *imagination* is more easily stimulated through physical activity than by thoughts alone (Kemp, 2012), in the setting provided by the Council of Care, the imaginative act, together with enacting, helps delve upon what might emerge if decision-making were to shift from an anthropocentric model to one of systemic care, where diverse beings and elements of nature collectively deliberate on the challenges affecting their shared environment.

To engage with the impacts of the oil spill, participants were assigned specific beings and invited to behave, walk, and act as those designated beings, with *acting* understood as the embodiment of a specific character. This embodiment creates a temporary ‘situational self’ (ibid.) reflecting upon a particular context; each person is considered as an organism existing in relation to one or more environments (Ingold, 2000). From this perspective, the whole-organism-in-its-environment is not a composite of separable parts such as body, mind, and culture, but a singular locus of creative process within a continually unfolding field of relationships (Zarrilli, 2007). Acting and enacting in this way enables participants to perceive and understand how a particular being or element relates to its environment in a more systemic and relational way.

I am considering here that acting through the embodiment of a fictional character allows one to perceive and understand the contextual environment. Although participants distorted or overly humanised the beings or elements through forms of anthropomorphism, it did not undermine their engagement with aesthetic experiences of animals and the environment. By doing so, they could consider the affinities and meeting points between human and nonhuman natures that allowed them to go through a process of decision-making aiming at collective and caring solutions. Such expressive qualities can deepen appreciation and open up new avenues that support respectful aesthetic valuing of animals and nature (Brady, 2014).

However, distinct from other accounts of understanding, the content of perception is not the same as the content of a picture or a text, we gain perception by active inquiry and exploration (Noë, 2004). Both Nöe and Ingold argue that it is impossible to separate perception, action and thought (Zarrili, 2007). According to them, to perceive is not merely to have sensations or to receive sensory impressions, it is to have sensations that one understands.

For Ingold (2000), this process of perceiving generates certain skills that are the capabilities of action and perception of the whole organism being situated in a rich structured environment. In such a discussion, there seems to exist a thin line between perception and understanding. For instance, in acting, perception should not be reduced only to subject feelings but to those sensations experienced that make some sort of sense, that is, that we understand the sensations being experienced according to a specific context (ibid.) which are accounted for through their aesthetic experience. This aligns with Poliseli’s processual account and Leonelli’s embodied account, in which specific skills are cultivated enabling the achievements of understanding.

Moreover, Noë (2004) examines two types of understanding without giving a clear distinction: sensorimotor understanding and conceptual understanding. According to him, perceptual experiences rely not only on the type and quality of the stimulus, but it is also shaped by the exercise of sensorimotor knowledge. Here, perceptual knowledge is practical knowledge (ibid.). Worthy note that the shape and feel of practice are not derived from or intrinsic to the sensations per se but rather are gained from what becomes an implicit sensory, embodied knowledge. In this sense, the Council of Care, by providing an acting activity of embodying elements of nature, allows the participants to understand the intricate, systemic, and wide-ranging impacts of the socioenvironmental problem of the oil spill in Siribinha.

## Towards an aesthetic account of scientific understanding

In previous sections, I introduced the *ClimArtLab Evolving Futures: Owning Our Mess* and the *Council of Care*. These research environments were inherently heterogeneous in their collaboration dynamics, disciplinary orientations, target phenomena, goals, and practices. Despite their dissimilarities, a common thread ran through both: the integration of aesthetic features in their research design. Because research environments in sustainability sciences are highly disunified, presenting a diversity of epistemic cultures (Schirone, 2024), epistemological debates on scientific understanding and aesthetic engagements need to consider not only literature coming from aesthetics of science but also, coherently, from environmental aesthetics. By interwoven these literacies while introducing both cases in previous sections, I uncovered how aesthetic experiences relate and foster scientific understanding in these research practices.

The main lesson from *ClimArtLab* and the *Council of Care* is that *aesthetic experiences enable achievements of understanding through embodied enactments of scientific knowledge*. In this view, understanding gained through aesthetic experiences is not passive, it emerges from an active interplay between theoretical knowledge, practical skills, and sensorial engagements. This triadic interaction, between subject (agents’ theoretical knowledge, practical skills, and sensorial engagements), object (target phenomenon or scientific information), and environment (research environment, practices, and goals), grounds a relational approach to aesthetic and understanding. *In contrast to traditional accounts that focus on aesthetic properties of theories or visual representations alone*,* my approach takes a relational turn*,* underscoring lived experiences*,* embodiment*,* and enactments as central to achievements of understanding*. Let me elaborate.

### Scientific understanding and aesthetic experiences

When aesthetic experience is examined in relation to environmental engagement, an integrated picture emerges: aesthetic experience, environment, and scientific understanding are mutually constitutive.

Accounts of **understanding through unification**, particularly those developed by Ivanova (2017a, 2020) emphasize that beauty is experienced in grasping relations among apparently disparate phenomena. Simplicity and harmony function as regulative ideals enabling scientists to comprehend relational structures. This resonates strongly with the climate-focused *ClimArtLab.* There, the water-energy-food nexus was not approached merely as a dataset to be simplified diagrammatically, but as a relational system to be experienced as interconnected. The embroidery practice in HOMONEXUS enacted unification materially: lines stitched together instantiated systemic relations rather than abstractly depicting them. The aesthetic experience of patterned interweaving facilitated an experiential grasp of nexus thinking, aligning with the idea that understanding consists in seeing how parts relate within a whole (Meynell, 2020).

The debate on **visualization and pictorial understanding** (De Regt & Dieks, 2005; De Regt, 2017; Meynell, 2018, 2020) further clarifies this connection. For De Regt, intelligibility is context-dependent, and visualizability is one among several tools that enhance understanding. Meynell, by contrast, regards pictorial representation as a necessary condition for understanding, since understanding requires “seeing” relations. In *ClimArtLab*, the glaciologist’s reliance on graphs exemplified De Regt’s contextual emphasis on visualization as enhancing intelligibility. Yet, the artists’ resistance highlighted a critical tension: when representation form does not align with experiential context, visualization may fail to generate understanding. Instead, embodied metaphor and dramaturgy offered alternative pictorial and imaginative scaffolds. The QR code sewn collectively, for example, functioned simultaneously as visual representation and participatory enactment, expanding pictorial understanding into embodied space.

The embodied account of understanding developed by Leonelli (2009) provides an additional bridge. Leonelli distinguishes theoretical, embodied, an integrated understanding in biological research, emphasizing the role of performative skills in grasping phenomena. The *Council of Care* operationalized such embodied understanding explicitly. By enacting rivers, mangroves, crabs, and policymakers in response to the Brazilian oil spill, participants cultivated performative and perceptual skills through which ecological relations became intelligible. Understanding did not emerge solely from propositional knowledge of the spill’s causes and impacts, but from embodied engagement within a structured environmental field. This aligns with Leonelli’s claim that different research contexts demand different forms of understanding, and that embodied competencies can be epistemically indispensable.

The role of imagination further consolidates this triadic relation. Breitenbach (2019) argues that the imaginative activities underpinning aesthetic experience in art are continuous with those supporting scientific understanding. Imagination enables scientists to unify phenomena under interpretive narratives and to explore conceptual possibilities. In GLACIER NEX US, the personification of a glacier functioned analogously to thought experiment (Brown, 2011; Murphy, 2020): an imagined dialogue explored relational and ethical consequences of climate change. The imaginative encounter did not replace scientific content; rather, it deepened relational comprehension by situating human-technology-nature dynamics within a dramatized scenario. Similarly, in the Council of Care, acting as nonhuman entities operated as an imaginative, goal-directed exercise that enabled participants to explore alternative decision-making models beyond anthropocentrism.

These examples also exemplify processual accounts of understanding, particularly that developed by Poliseli (2020). Understanding is achieved in degrees, is sensitive to complexity, and depends on imagination and knowledge selectivity. Both the climate nexus and the oil spill represent highly complex socioecological phenomena. In such contexts, reduction to simplified representations may obscure systemic relations. The aesthetic practices described (collective embroidery, performance, enactment) facilitated gradual attunement to complexity. Participants learned to filter relevant from irrelevant information through experiential engagement, illustrating Poliseli’s claim that greater understanding equips one with better epistemic selectivity. Imagination and embodied participation functioned as mediators in navigating complexity, consistent with Breitenbach (2019) and Stuart (2018).

Across these directions (unification, visual, pictorial, embodied, imaginative, and processual) a common structure becomes visible. Aesthetic experience is not merely an evaluative overlay on scientific practice; it is also a mode through which (socio)environments become intelligible. The environment, whether conceptualized as a climate system or oil-affected ecosystem, provides the relational complexity that calls for unification. Aesthetic practices (visual, embodied, imaginative) mediate access to that complexity (Ivanova, 2020). Scientific understanding, in turn, emerges not solely from abstract representation, but from situated engagement within an environmental field structured by perception, action, and interpretation.

Thus, rather than occupying an auxiliary role, aesthetic experience operates as a connective tissue between environmental reality and epistemic achievement (Potochnik, 2017; Elgin, 2018, Ivanova, 2020). It enables scientists and collaborators to grasp relations, navigate complexity, and cultivate the skills necessary for understanding phenomena whose systemic character resists reduction. In this sense, aesthetic experience, environment, and scientific understanding form an integrated triad: environments structure aesthetic engagement; aesthetic engagement mediates epistemic access; and scientific understanding crystallizes through this relational process.

### Aesthetic experiences and other tools enabling understanding

It is hard to deny that visual theories are often regarded as more intelligible than abstract ones in fostering understanding, as many scientists prefer visual reasoning in the construction of explanations (De Regt, 2002). This is similar to paleontology, evolutionary biology, botany, and so on. Poliseli et al. (2022) has argued that visualization played an important role in the scientific practice of mechanistic model building in bee biodiversity conservation, through the creation of diagrams, mechanisms, and graphs. If we consider modelling from the artefactual perspective where models are artifacts (Knnuttila, 2005) mediating between theory and the world (Morgan & Morrison, 1999), we can understand models as visual artifacts. Thus, the development of several models during heterogeneous research environments is already expected as well as its epistemic role of providing understanding, as defended by De Regt (2002) and Meynell (2020).

However, in the interdisciplinary *ClimArtLab - Evolving Futures: Owning Our Mess*, visual tools did not possess the same epistemic advantage, opposing De Regt, and Meynell argument of scientific understanding in disciplinary practices. Instead, *metaphors* were prominent in fostering understanding. Besides the controversies of using scientific metaphors (Keller, 1995, Taylor & Dewsbury 2018, Reynolds, 2022), metaphors in science are vastly employed because understanding an unfamiliar phenomenon is often assisted by analogy reasoning (Hesse, 1963; Bailer-Jones, 2002), allowing one to make concrete connections between abstract concepts and everyday experiences (Taylor & Dewsbury 2018). Although they might obscure some processes, they can also show unexpected dynamics or operate in more underdetermined ways which opens up space for multiple lines of reading and re-reading (Keller, 2000).

In the *ClimArtLab*, metaphors were useful tools in advancing understanding of Climate Change because of their aesthetic characteristics, that is, to experience a representational symbolic system in and from a different domain (Ramirez, 2003). Understanding the water-energy-food nexus through a process of embroidery of a QR-code was only possible because metaphors are not only adornments. They constitute an integrative language (ibid.) allowing the development of thought processes and conceptual understanding giving meaning to knowledge or perceptions from one domain to another (Taylor & Dewsbury, 2018). If we consider that the nature of human cognition is metaphorical, all knowledge will emerge from experiences that are socially and physically embodied (Lakoff & Jonhson, 1980). This resonates with one of the artists in the *ClimArtLab*:

This is connected to the idea of climate change. The idea that we want to change but we still keep doing things that we know are bad, experiencing this kind of dissonance. So, what could be a possible approach to reduce this cognitive dissonance? Learn by doing. I was thinking of a collective ritual, where it is possible to share knowledge and experiences, with no hierarchy, no pressures, and reducing the dissonance. There is a very interesting quote from Confucius that resumes what I think “We hear, we forget. And then we see, and we remember. But when we do, we understand”. This is to me, a basic principle. Slowing-down activities can bring the introspection we need to process the information and reduce our inner dissonance. But how to change? The change is already there somehow. It cannot be perceived straight away but it is already happening. When we plant a seed we don’t go there removing the soil each hour to see if it’s growing. We water it and wait until the plant manifests itself. So it’s somehow an act of trust. Sewing is a very interesting activity and it can be everywhere as it can be a very political activity. Thinking about sewing, this quote also speaks to me “The great change we are willing to see in the world passes literally through our hands and body” (Artist, *edited by author*).

Meanwhile, in the transdisciplinary *Council of Care*, an arts-based methodology was developed in order to foster embodied understandings about the impacts of a socioenvironmental problem, the oil spill. According to Brown (2003), the embodied understanding of conceptual frameworks and theoretical models is the same embodied understanding of the world that is unconsciously deployed in day-to-day activities and social interactions. Understanding through aesthetics is attuned to a context and is especially well-equipped as to adjust to our sensory and cognitive environments, therefore, it is a mode of cognition grounded in the perceptual senses (Saunders, 2021). Aesthetic experiences such as those adopted in the *Council*, and beyond, can serve as opportunities to modulate, soothe, enhance, rewrite, explore, feel, forget, or merely reflect upon distinct aspects such as memories, interests, desires, or habits in a socially situated, extended, intersubjective, and embodied context (Vara Sanchez, 2022).

Thus, imagination, metaphors, arts, and others are powerful tools when framing socio-environmental issues, such as biodiversity loss, oil spill, climate change, etc. It can influence the perception of urgency, and risk, as it can foster intrinsic motivation towards change by acting on pre-existing cognitive schemas and prompting affective responses (Thibodeau & Boroditsky, 2013; Galafassi et al., 2018a, b). In the *ClimArtLab*, the embroidery of a QR code allowed this metaphor to shape the mind, structure the experience, and potentially influence behaviours by reflecting on slowing down water, energy, and food consumption (see Lakoff & Johnson, 1980). In the *Council of Care*, acting and enacting elements of nature was an invitation to reflect towards practical uses of conservation.

The aesthetic experiences partaking in scientific understanding is inherently diverse. This is because aesthetic experiences are not singular or belong to one particular type of experience. There are many types of aesthetic experiences (Pole, 1955) that are also not detached from other non-aesthetic experiences (Vara Sanchez, 2022), which grants them a multimodal and prismatic disposition (Saunders, 2021). Therefore, *aesthetics becomes a key component in achievements of understanding as it can adapt to (inter)disciplinary and comparative forms of inquiry.* It not only shapes how knowledge is produced, disseminated, and understood in written propositional forms, but can also extend to other contexts and perceptual regimes involving diverse material, intellectual, social, and semantic resources (Saunders, 2021). This includes the kinds of knowledge emerging from sustainability sciences, inter- and transdisciplinary research practices, as well as arts and sciences such as those conveyed in the *ClimArtLab* and the *Council of Care*.

### The environment as a constitutive field in aesthetics and understanding

Within the relational account of understanding, the environment is not treated as a neutral backdrop for aesthetic episodes but as a constitutive dimension of aesthetic experience itself. Across major theoretical approaches, it functions simultaneously as epistemic object, relational medium, and atmospheric condition, structuring perception, affect, imagination, and evaluation (Carlson, 1998). This conceptual framework gains particular clarity when examined through transdisciplinary practices addressing climate change and biodiversity loss.

Cognitive approaches, most prominently associated with Allen Carlson (1981, 2005), argue that appropriate appreciation of environments depends on relevant knowledge frameworks (ecological, geological, or scientific). Similarly, Turner (2019), looking at the deep past in paleoaesthetics, has also argued that because environments are historically constituted, full aesthetic appreciation of landscape and fossils depends on scientific knowledge of their deep-time histories. Environmental features such as glacial retreat or ecosystem dynamics become aesthetically appreciable when grasped through informed categorization, here aesthetic experiences are merged with epistemic inquiry. Hence, aesthetic judgment is structured by environmental properties understood under appropriate scientific descriptions. Visual models and graphs of temperature rise or sea-level change exemplify this approach: intelligibility is secured through visualizable (De Regt, 2017), understanding is secured through idealizations (Potochnik, 2017), and the environment appears as an epistemic object whose complexity can be rendered legible (Ivanova, 2020).

However, transdisciplinary climate collaborations reveal the limits of reducing environmental intelligibility to diagrammatic clarity. In *ClimArtLab*, artists resisted what they perceived as the ‘diagrammification’ of climate knowledge. While scientific representations present climate systems as extended data patterns, they may fail to situate perceivers within the lived relational field of those systems. The environment, understood as a dynamic nexus of water, energy, and food systems, resisted compression into static graphical form. This can be related to Potochnik’s (2017) discussion that idealizations in science, and its visual representations, are inherently distinct according to its specific scientific goals.

This shift toward lived relationality resonates with engagement-based theories, where aesthetic experience is participatory and immersive (Berleant, 2017). Perceiver and (natural and human-modified) environment are dynamically interrelated within a shared field. In the artistic intervention HOMONEXUS, collective embroidery enacted the interconnectedness of socioecological systems through embodied, rhythmic engagement. Sewing functioned both metaphorically and materially: environmental complexity was not simplified into representation but enacted through patterned interweaving. The environment appeared not as a distant object but as a lived relational process.

Similarly, GLACIER NEX US staged a dialogical exchange between a glaciologist and a personified glacier. The glacier was transformed from an object of detached measurement into a participant within a shared world. Aesthetic experience here reorganized the human-environment relation, rendering interdependence, technological thriving, and ecological disappearance experientially salient. The environment emerged as a relational partner rather than external referent.

Bohme’s (year) atmospheric work help further clarify this dynamic. Atmospheres are neither purely subjective states nor objective properties; they arise in the relational space between perceiver and environment. Environment configurations (light, sound, spatial arraignment, materiality) generate affective tonalities that condition experience prior to explicit judgment. In *ClimArLab*, music, collective crafting, and dramaturgy produced shared affective spaces in which climate complexity was sensorially enacted rather than merely conceptualized. Understanding emerged through experiences of feeling before propositional articulation.

A parallel structure characterized the Council of Care where participants embodied rivers, mangroves, crabs, policymakers, and fishers. The environment ceased to function as an external problem-space and instead became the very medium of deliberation. Through embodied enactment, participants navigated systemic interdependencies from within an ecological field.

This process aligns with enactive accounts of perception, particularly those of Noë (2004) for whom perception is an active sensorimotor engagement with the world. Acting as a mangrove or ocean current did not merely symbolize ecological entities, it cultivated practical, perceptual skills through which ecological embeddedness became experientially intelligible. The environment structured possibilities for perception and action, blurring the distinction between sensing and understanding.

Taken together, environmental aesthetics and these research practices converge on an unified claim: the environment is not simply what aesthetic experience is about, it is what aesthetic experience happens within and through. It provides the structured complexity that calls for interpretation, the relational field within which embodied engagement unfolds, and the atmospheric conditions that modulate affective and evaluative response. In contexts of climate change and biodiversity loss, aesthetic experience thus operates as a mode of environmental attunement. Rather than reducing complexity to data alone, these practices situate perceivers within systemic interdependence, rendering environmental crises cognitively intelligible, relationally immediate, and normatively compelling.

### Relational character of aesthetics and understanding

Understanding complex social-ecological impacts is not straightforward. Scientific knowledge about these issues often fails to foster understanding among those without a strong scientific background, such as those encountered in specific disciplinary research environments. While this was evident in the *ClimArtLab*, in the *Council of Care*, the aesthetic experiences allowed participants to engage with complex-socioenvironmental impacts in ways that connected directly to their daily lives and their lived experiences during the oil spill. This relational aesthetic experience challenges dichotomies such as subject and object, human and non-human, helping participants to recognize themselves as part of the environment, interacting with its elements while understanding its interconnectedness (Berleant, 2017).

These relational aesthetic experiences challenge the duality of object-subject relationship, in the same way that the aesthetic account of understanding challenges the duality of understanding from object-subject to object-subject-environment. Aesthetic experiences do not need to be passive acceptances of what an object presents nor purely subjective responses rooted solely in the self (Saito, 2022). They can arise through relationality and interdependence: there is no aesthetic perceiver without an object of perception, and no perceptual object without a perceiver (ibid.). The same relational dynamic can be applied to understanding. *Understanding does not need to be manifested in sentences*,* it can equally be expressed in suitable terminology*,* insightful cues*,* effective nonverbal symbols*,* and so on* (Elgin, 1993). This validates the attunement of the embodying of something (either beings of nature in the *Council of Care* or the water-food-energy nexus in the *ClimArtLab*) through distinct practices of acting or embroidering, allowing the understanding of socioenvironmental problems to emerge from these embodied and enacted experiences.

Considering that aesthetic experiences are a subset of perceptual experiences distinguished through dynamic relations rather than object features and attitudes alone (Brincker, 2015), the experience provided by ecological art, in general, relies on sensory, perceptual, imaginative, and emotional interactions, which allows one to generate particular dispositions toward understanding the natural world as it reflects upon the distribution of agency during interactions (Brady et al., 2018). The integrated role of perception, imagination, cognitive and scientific appraisals shape these aesthetic experiences (Currie et al., 2014) and by developing ever-novel ways of acting in, interacting with, and making sense of the world, aesthetic experiences in sustainability research environments helps enrich human cognitive embodiment to better understand complex socio-ecological and conservation challenges. This capacity to intervene in the phenomenon whether by performative skills or theoretical knowledge is explored by Leonelli (2009) on *integrated understanding* when dealing with scientific understanding in biological research practices, and Elgin (1993) when speaking about understanding in physics.

According to Currie (2023b), there is no good reason to assume that the subjectivity of aesthetic judgments is disconnected from or harmful to scientific judgment. If epistemology and aesthetic shape each other, then some mutual influence is expected and not problematic. Our scientific judgments will not be aesthetically neutral, and although Currie (ibid.) argues that they are acceptable as long as aesthetic values do not solely determine scientific outcomes, I argue that aesthetic experiences, given appropriate relational circumstances, can inform not only scientific outcomes but also provide scientific understanding. Let me use *imagination* to elaborate.

*Imagination* is a key aspect of aesthetic experience and according to the processual account of understanding, it functions as an epistemic mediator for achievements of understanding. Imagination is considered as a higher mental action (Tateo, 2020) capable of testing propositions by seeing whether they are conceivable through mental images, thought experiments, mental models, and so on. This is only possible when imaginative experiences are not dismissed as epistemologically insignificant, there are a variety of ways one can use imagination. According to Todd (2020),

The intentional object of imagining, it seems, is not images. Images are mere, in the words of Kind, a type of ‘mental paint’ with which the imagination takes its intentional objects, the objects that are imagined. We employ images to help us imagine but we - so to speak - see right through them to the things we are actually imagining (p. X).

Breitenbach (2019) acknowledges that the same type of understanding that uses imagination in scientific practices is the same during the appreciation of an artwork. Both imagination in science and art are fictions, vehicles for exploration and discovery, capable of providing contexts in which features may be demarcated, their interplay examined, and their implications drawn out (Elgin, 1993). If constraints are delineated according to real-world facts (or phenomenon-oriented), then this epistemic procedure of imagining is adaptable to scientific experimentation (Kind, 2018). Hence, this cognitive sense-making of the phenomenon and environment through imagination and experiments becomes an embedded and embodied bodily experiences evolved from perceptually-oriented action and action-oriented perception, which allows the use of the imaginative process in which meaning and understanding are linked to sensorimotor experience, as well as the conceptual tools such as visual, verbal, mathematical, thoughts, musical, and others (Wrigth-Carr, 2019).

This is consonant with *processual understanding* where the relationship between subject-object-environment is constantly adjusted. The object (i.e. phenomenon) dictates which methodological strategies and conceptual frameworks (i.e. research environment) will be adopted by the scientist (i.e. subject). Once visual tools aid in achieving understanding of the target phenomenon, the scientist (i.e. subject) adjusts research goals (i.e. object) and research strategies (e.g. imagination) accordingly (i.e. environment). *Distinct from the processual account that only acknowledges imagination as enabling understanding*,* a relational account of understanding considers the entire spectrum of aesthetic experiences with the potential of facilitating understanding.* This is because there are certain properties, qualities, experiences, values, or judgments associated with perceptions arising from the sensory, imaginative, and emotional dimensions between the subject, object and environment (Brady et al., 2018) that can be useful to the development of scientific knowledge and achievements of understanding.

If scientific knowledge is produced through an iterative, interactive process in which investigators and their objects of study mutually shape and constrain one another (Jasanoff, 2004), and if scientific practices involve finely attuned aesthetic judgments calibrated for scientific purposes (Currie 2023a), then the relational role of aesthetic experiences in achieving understanding should not surprise us at all. Instead, it should alert us to the fact that aesthetic experiences are not a peripheral feature of scientific understanding, but one indispensable part of.

## Conclusion

Despite increasing efforts to incorporate aesthetic features in accounts of scientific understanding, there are still several challenges. First, these efforts often remain confined to disciplinary contexts, overlooking how understanding is shaped within interdisciplinary settings (where aesthetic and epistemic practices intersect more intricately and knowledge is situated in broader academic, cultural, and societal frameworks). Second, they tend to restrict understanding to experts by framing it solely in terms of complex propositional or pictorial knowledge, neglecting the need for inclusive forms of communication socio-ecological problems in public-facing contexts. And third, they frequently reduce aesthetic engagements either to focus on aesthetic features (thereby missing its inherent heterogeneity across scientific objects, products, practices, collaborations, communication, and policy interfaces) or to static properties of theories and models (neglecting its dynamic nature).

Using an empirically oriented and socially engaged approach, I examined the aesthetic experiences present in two transdisciplinary research environments from sustainability sciences, the *ClimArtLab - Evolving Futures: Owning Our Mess* and the *Council of Care*. I outlined a relational account of aesthetic experiences in scientific understanding, showing that this relationship is not only psychological and contingent to scientific practices but also embodied and enacted, continually shaped by the interplay between the subject (scientists’ theoretical knowledge, practical skills, sensorial inputs, and emotions), the object (target phenomenon), and the surroundings (research environment, practices, and goals).

A relational account of aesthetics in scientific understanding, one in which understanding is achieved through the embodied enactment of scientific knowledge, encourages us to reconsider the role of aesthetic tools not merely as epistemically pleasing. The relational aspect between practical skills, theoretical knowledge, target phenomenon, and scientific practices becomes especially significant to understand complex socio-environmental problems in transdisciplinary settings. While this account holds that scientific understanding is a process of embodied enactment of scientific knowledge through aesthetic experiences, further work is needed to address questions such as how 4E cognition relates to scientific understanding in sustainability research environments, or how phenomenological theories might illuminate understanding beyond disciplinary academic contexts.

This paper recognizes aesthetic experience not merely as a source of understanding, but as a robust epistemic tool across diverse practices and research environments capable of framing, explaining, and deepening our understanding of urgent socio-environmental challenges, from biodiversity loss and natural disasters to climate change and planetary health. By bridging asthetics of science and environmental aesthetics, this paper opens a broader agenda: one that situates aesthetic experience at the core of epistemic practices in addressing socio-environmental crises.

## Data Availability

Raw data were generated at Konrad Lorenz Institute for Evolution and Cognition Research (KLI), and Wageningen University & Research (WUR). Derived data supporting the findings of this study are available from the corresponding author on request.

## Footnotes

1 Given the breadth and terminological proliferation within the aesthetic lexicon, I proceed from the assumption that aesthetic experiences constitute a subset of perceptual experiences, differentiated not primarily by intrinsic object properties or discrete mental attitudes, but by dynamic relational process (Brincher, 2015). At the same time, I adopt the broadly accepted view that aesthetic responses can ground aesthetic judgments insofar as they enable the ascription of value (Brady, 2003).

2 Since McAllister’s pioneering contributions, a growing body of literature has emerged to clarify the complex relationship between aesthetics and science. Due to reasons of space, significant discussions are not included in this article. Some works worthy of mention are Chandrasekhar (1987), Jones and Galison (1998), Rothenberg (2012), Shimamura and Palmer (2014), Bueno et al. (2017), Ivanova and French (2020), and Ivanova and Murphy (2023).

3 For disambiguation, I understand “environment” as broadly constructed encompassing both natural and social dimensions that shape our surroundings. In the same line, “research environment” is conceived broadly (overall in inter- and transdisciplinary contexts) extending beyond academic and institutional settings to include societal and civic contexts, as well as natural and human-modified environments (such as ecosystem, urban spaces, and so on).

4
https://climartlab.space/.

5
https://www.geos-project.org/.
